# Kinesin 6 Regulation in *Drosophila* Female Meiosis by the Non-conserved N- and C- Terminal Domains

**DOI:** 10.1534/g3.117.300571

**Published:** 2018-03-09

**Authors:** Arunika Das, Jeffry Cesario, Anna Maria Hinman, Janet K. Jang, Kim S. McKim

**Affiliations:** Waksman Institute, Rutgers, the State University of New Jersey, NJ-08854

**Keywords:** Kinesin 6, Meiosis, Drosophila, chromosome segregation, microtubule, oocyte

## Abstract

Bipolar spindle assembly occurs in the absence of centrosomes in the oocytes of most organisms. In the absence of centrosomes in *Drosophila* oocytes, we have proposed that the kinesin 6 Subito, a MKLP-2 homolog, is required for establishing spindle bipolarity and chromosome biorientation by assembling a robust central spindle during prometaphase I. Although the functions of the conserved motor domains of kinesins is well studied, less is known about the contribution of the poorly conserved N- and C- terminal domains to motor function. In this study, we have investigated the contribution of these domains to kinesin 6 functions in meiosis and early embryonic development. We found that the N-terminal domain has antagonistic elements that regulate localization of the motor to microtubules. Other parts of the N- and C-terminal domains are not required for microtubule localization but are required for motor function. Some of these elements of Subito are more important for either mitosis or meiosis, as revealed by separation-of-function mutants. One of the functions for both the N- and C-terminals domains is to restrict the CPC to the central spindle in a ring around the chromosomes. We also provide evidence that CDK1 phosphorylation of Subito regulates its activity associated with homolog bi-orientation. These results suggest the N- and C-terminal domains of Subito, while not required for localization to the central spindle microtubules, have important roles regulating Subito, by interacting with other spindle proteins and promoting activities such as bipolar spindle formation and homologous chromosome bi-orientation during meiosis.

During meiosis I, pairs of homologous chromosomes segregate from each other, resulting in a reductional division. Bi-orientation, which is the arrangement of homologous centromeres toward opposite poles, is a critical part of metaphase I since it establishes how homologous chromosome pairs segregate at anaphase I. Errors in this process are a leading cause of infertility and birth defects in humans (Nagaoka *et al.* 2012). Indeed, the fidelity of meiosis is fundamentally important to all sexually reproducing organisms and depends on the formation of a bipolar spindle and accurate chromosome bi-orientation. Oocytes in many animals accomplish these tasks without centrosomes, which normally organize the poles of a spindle. Oocytes have mechanisms in place to compensate for the absence of centrosomes, but this feature may also contribute to their relatively high error rate (Dumont and Desai 2012; Radford *et al.* 2016).

In *Drosophila*, a robust metaphase I central spindle, composed of an array of microtubules in anti-parallel overlap, plays an important role in oocyte spindle organization and homolog bi-orientation (Jang *et al.* 2005; Radford *et al.* 2015). The *Drosophila* meiotic central spindle array has features in common with the anaphase midzone in mitosis, which has a role in defining the cleavage furrow for cytokinesis (D’Avino *et al.* 2015). In fact, the Drosophila meiotic central spindle contains several proteins which are required for midzone function in anaphase of mitosis, such as the centralspindlin complex (RacGAP50C and Pavarotti/ MKLP1) and Subito (MKLP2), FEO/PRC1 and KLP3A/KLP4 (Williams *et al.* 1997; Jang *et al.* 2005; Riparbelli and Callaini 2005; Das *et al.* 2016). Interestingly, although the mitotic midzone forms between segregating chromosomes at anaphase, the meiotic central spindle assembles around the chromosomes prior to segregation during prometaphase I. This represents a situation where similar structures may form in meiosis and mitosis, but have unique functions specific to oocyte acentrosomal spindles. The central spindle during meiotic metaphase may be a conserved feature of oocytes that compensates for the absence of centrosomes (Dumont and Desai 2012). For example, the meiotic chromosomes of mouse oocytes arrange in a “prometaphase belt”, which is in the center of the forming spindle, prior to bi-orientation at metaphase I (Kitajima *et al.* 2011).

Subito is a kinesin 6 that is required for assembling the meiotic central spindle, metaphase I spindle bipolarity, proper localization of the chromosome passenger complex (CPC) and, as a result of these functions, promoting accurate chromosome segregation (Giunta *et al.* 2002; Colombié *et al.* 2008; Radford *et al.* 2012). In mammalian cells, the Subito homolog MKLP2 is required for cytokinesis (Gruneberg *et al.* 2006; Kitagawa *et al.* 2013). MKLP2 is required for translocation of the CPC from the centromeres to the spindle midzone and this interaction is negatively regulated by Cdk1 (Hümmer and Mayer 2009; Kitagawa *et al.* 2014). It is unclear, however, how the localization and activity of Subito functions to promote chromosome segregation during meiosis I and how it is restricted to the central spindle in meiosis. It is not known what targets a kinesin 6 like Subito to the central spindle, nor how it interacts with the CPC and if this interaction is regulated by Cdk1.

All kinesins have a highly conserved ATPase-containing motor domain. In contrast, most kinesins, including the kinesin 6 family, have more highly diverged sequences on one or both sides of the motor domains that may be important for their regulation and unique activities. For example, during *Drosophila* oogenesis, the central stalk region of Pavarotti is required for localization to ring canals and for binding to spindle midzone microtubules (Matuliene and Kuriyama 2002; Minestrini *et al.* 2002). The C-terminal domain of MKLP2, has been shown to be important for binding Mad2 (Lee *et al.* 2010) and regulates interaction with spindle microtubules (Kitagawa *et al.* 2014). The C-terminal domain of MKLP2 also contains a lipid association motif and is a target of Aurora B kinase (Fung *et al.* 2017). Interactions between the C- and N-terminal domains of some kinesins may be an auto-inhibitory mechanism (Verhey and Hammond 2009). Indeed, deletion of the N-terminal domain of Subito causes ectopic spindles to appear that are detached from the chromosomes (Jang *et al.* 2007). These results suggest that kinesin 6 is negatively regulated through its N-terminal domain to restrict oocyte spindle assembly around the chromosomes. Furthermore, while most kinesins are considered to contain either N-terminal (kinesins 1-12) or C-terminal (kinesin 4) motors (Verhey and Hammond 2009; Welburn 2013), our evidence suggests that the kinesin 6 motor domain is centrally located between two non-motor domains (Jang *et al.* 2007), similar to the kinesin 13 family.

We predict that the regulation of kinesin 6 in mitosis and meiosis may reside in the diverged N- and C-terminal regions. In this study, we have investigated the contribution of the N- and C-terminal domains and putative CDK1 phosphorylation sites of Subito to spindle assembly and motor localization. The N-terminal domain contains antagonistic positive and negative regulators for localization of Subito to microtubules. Some of the N-terminal domain conserved in Dipterans, however, are not required for meiosis but are crucial for embryonic development. The analysis of phosphomimetic mutations suggests preventing CDK1 phosphorylation of Subito is important for function but not localization during meiosis. This study has identified domains within both the Subito N- and C-terminal domains that, while not required to specify localization to the central spindle, are required for specific functions such as restricting localization of the CPC component INCENP to the central spindle.

## Materials and Methods

### Generation and initial analysis of transgenic lines

A full-length derivative of *subito* was amplified by PCR. The clone was verified by sequencing and then cloned into pENTR2B vector (Gateway). The fragment was then recombined using Clonase (Invitrogen) into the pPHW vector which encodes three copies of the HA epitope at the N-terminus of the coding region in a pUASP backbone (Rørth 1998). The *sub^Δ(1-21)^* construct was created by cutting the wild-type *subito* pENTR2B construct with *Bam*HI and *Eco*RI. The resulting 1600 bp fragment was re-cloned back into pENTR2B. This pENTR2B clone and wild-type *Subito* pENTR2B were both cut with *Eco*RI resulting in a 3712 bp fragment and a 796 bp fragment respectively. After CIP treatment, these fragments were ligated to each other, resulting in a *subito* clone missing the first 21 amino acid, but maintaining the same open reading frame. The remaining deletions and amino acid substitutions were created using the Change IT mutagenesis kit (USB) and the appropriate primers on the wild-type *subito* clone in pENTR2B.

To measure fertility and chromosome segregation during meiosis, females were crossed to *y w/B^s^Y* males. The non-disjunction frequency was calculated as 2(B^S^ ♀+ B^+^ ♂) / [B^+^ ♀+ B^S^ ♂+ 2(B^S^ ♀+ B^+^ ♂)]. Ovary protein levels were assayed by Western blot. Whole ovaries were dissected from yeasted females in PBS and then ground and boiled in SDS gel loading buffer. Protein from ∼2 to 3 ovaries was loaded per lane. The primary antibody was rat-anti HA “high affinity” (Roche, clone 3F10) used at 1:5000; the secondary HRP-conjugated antibodies (Jackson Labs) were used at 1:5000. The secondary was detected using ECL reagents (Amersham, Piscataway, NJ).

### Antibodies and immunofluorescent microscopy

Stage 14 oocytes were collected from 50 to 200 3 to 4 day old yeast fed non-virgin females by physical disruption in a common household blender (McKim *et al.* 2009) (Theurkauf and Hawley 1992). The oocytes were fixed in modified and 100 mM cacodylate/8% formaldehyde fixative for 8 min and then their chorion and vitelline membranes were removed by rolling the oocytes between the frosted part of a slide and a coverslip. For immunofluorescence rolled oocytes were extracted in PBS/1% Triton-X-100 for 1-2 hr and blocked in PBS/0.1% Tween-20/0.5% BSA (PTB) for an hour and then antibodies were added. For FISH, rolled oocytes were stepped into 20%, 40% and 50% formamide solutions followed by 5 hr incubation in 50% formamide at 37°. Oocytes were incubated with the FISH probes at 91° for 3 min and then put into the 37° water bath overnight. Oocytes were stepped out of formamide and blocked in PTB for 4 hr before addition of antibodies (Radford *et al.* 2012).

Oocytes were stained for DNA with Hoescht 33342 (10µg/ml) and for microtubules with mouse anti-α tubulin monoclonal antibody DM1A (1:50), directly conjugated to FITC (Sigma, St. Louis) or rat anti-α tubulin monoclonal antibody (1:75) (Millipore). Other primary antibodies were rat anti-Subito antibody (used at 1:75) (Jang *et al.* 2005), rat anti-HA (Roche, clone 3F10) (1:25), rat anti-INCENP (1:500) (Wu *et al.* 2008) and rabbit anti-CID (Active Motif, 1:1000). These primary antibodies were combined with either a Cy3 or Cy5 secondary antibody preabsorbed against a range of mammalian serum proteins (Jackson Immunoresearch, West Grove, PA). FISH probes used were to the AACAC repeat (2^nd^ chromosome) and dodeca repeat (third chromosome) and 359 probe (X chromosome). Oocytes were mounted in SlowFade gold (Invitrogen). Images were collected on a Leica TCS SP2, SP5 or SP8 confocal microscope with a 63x, NA 1.3 or 1.4 lens respectively. Images are shown as maximum projections of complete image stacks followed by merging of individual channels and cropping in Adobe Photoshop (PS3).

For detecting protein levels by Western blot, whole ovaries were dissected from yeasted females in PBS and then ground and boiled in SDS gel loading buffer. Protein from ∼2 to 3 ovaries or embryos were loaded per lane. The primary antibody was rat-anti HA “high affinity” (Roche, clone 3F10) used at 1:5000 or rat anti-Subito at 1:2000. The secondary HRP-conjugated antibodies (Jackson Labs) were used at 1:5000 and were detected using ECL reagents (GE).

### Data and Reagent Policy

**Figure 1:**
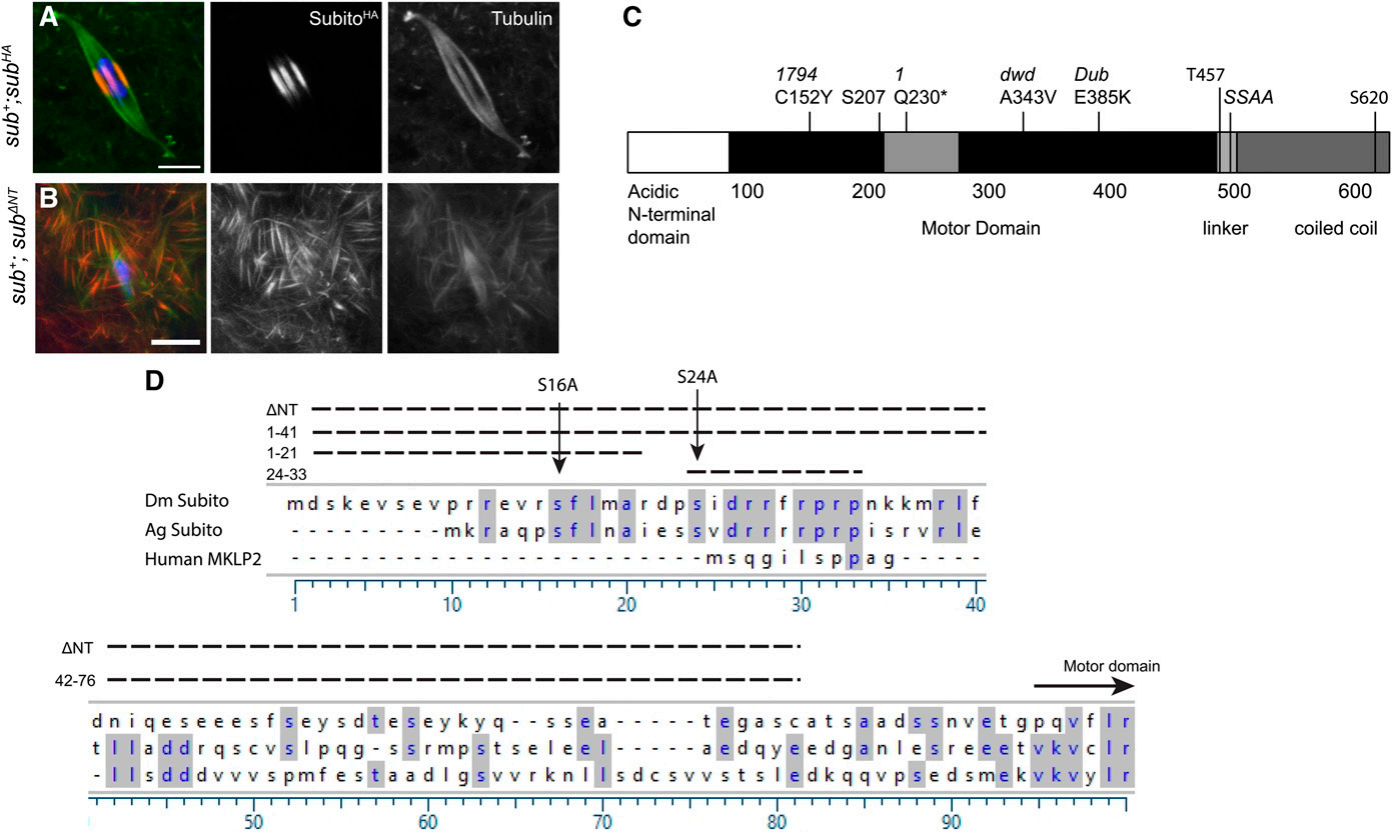
Ectopic bundling by Subito^ΔNT^ and alignment of N-terminal domain. A. Wild type oocytes expressing *sub^ΔNT^*, stained with DNA (blue), HA (red) and Tubulin (green) and single channels are shown in white. Scale bars are 5µm. B. Ectopic bundling of microtubules can be seen in the ooplasm with SUB^ΔNT^ localized to them. C. Schematic of Subito structure. The black is the conserved motor domain. The light block within is the sequence unique to the kinesin 6 family. Mutations in this study and others are shown above and the domains are named below. The three predicted proline-directed CDK1 phosphorylation sites are shown by the amino acid locations (S207, T457 and S620). D. Alignment of the Subito N-terminal domain in *Drosophila melanogaster*, *Anopheles gambiae* and human (MKLP2). Deletions made are also indicated above the sequence and the two conserved serine sites that may be involved in phospho-regulation of the protein.

**Figure 2:**
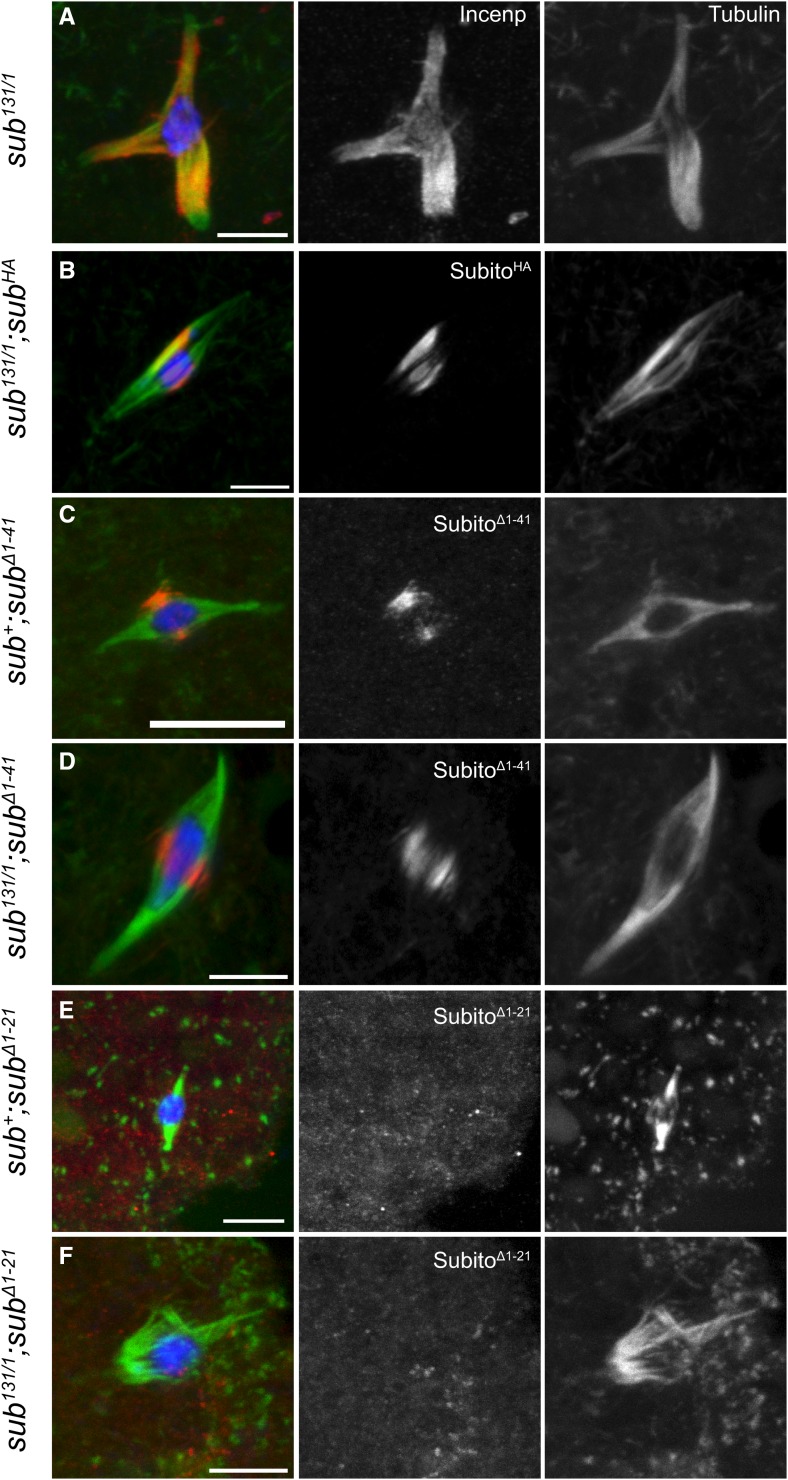
The first half of the N-terminus regulates localization to microtubules and robust central spindle assembly. Oocytes stained for DNA (blue), INCENP or HA (red), tubulin (green). The single channel images are for INCENP, HA and tubulin are shown in white. Scale bars are 5µm. A. *sub^131^*^/^*^1^* null mutant oocyte showing a bipolar spindle lacking a central spindle with diffuse localization of INCENP. B. Expression of wild-type full length Subito^HA^ in a *sub^131^*^/^*^1^* oocyte. C-F. Localization of indicated transgenes in *sub^+^* or *sub^131/1^* backgrounds. Subito^Δ(1-21)^ does not localize as seen in E and F. (n= 30, 8, 22, 13, 9 and 4 respectively for each genotype).

**Figure 3:**
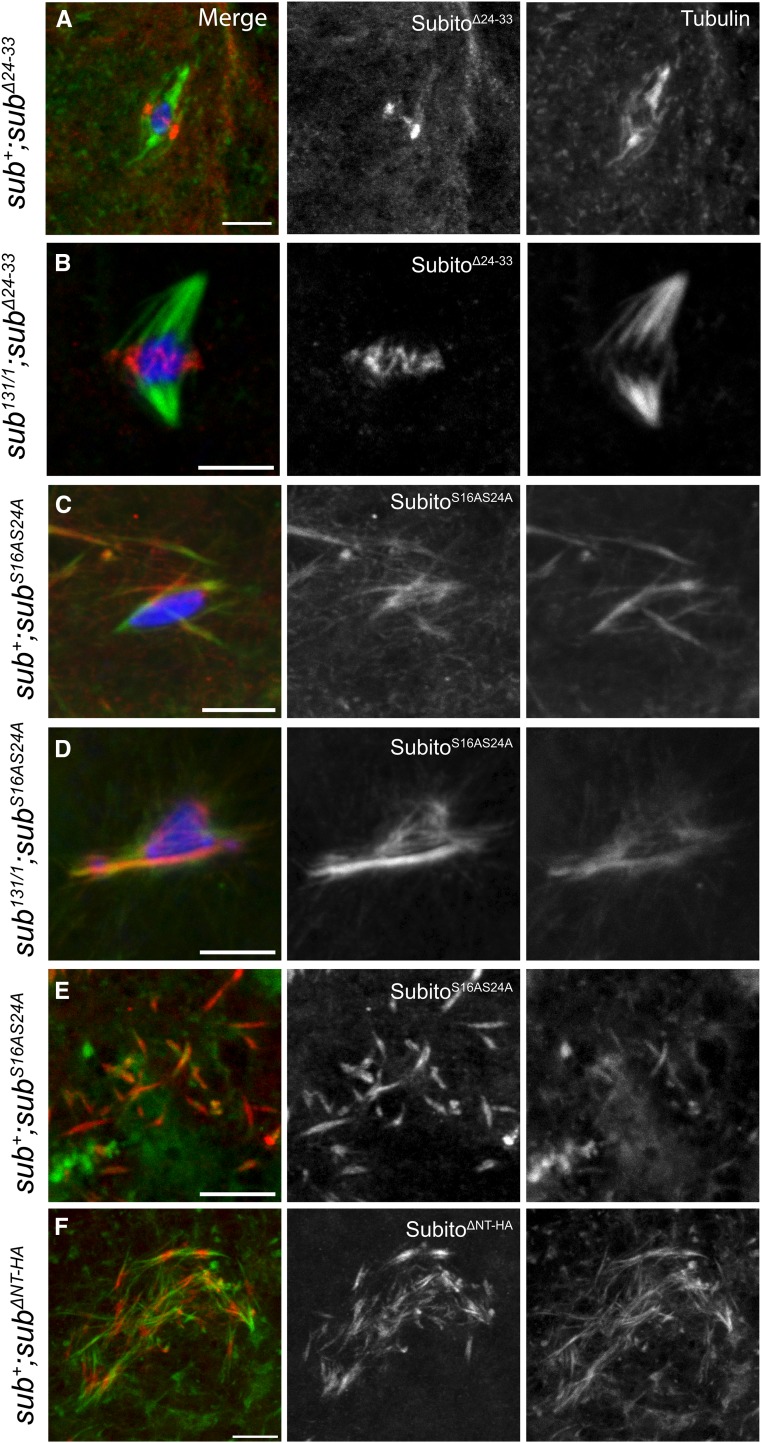
Conserved domains in the N-terminus are dispensable for meiosis but regulate ectopic microtubule bundling in ooplasm. Oocytes stained for DNA (blue), HA (red), tubulin (green). Single channels for HA and tubulin are shown in white. Scale bars are 5 µm. A, B. Localization of Subito^Δ(24-33)^ in *sub^+^* or *sub^131^*^/^*^1^*background. Subito^Δ(24-33)^ shows normal localization. C, D. Localization of Subito^S16AS24A^ in *sub^+^* or *sub^131^*^/^*^1^*background. (n= 21, 5, 22 and 5 respectively for panels A-D). E, F. Examples of ectopic microtubule bundling in oocytes expressing Subito^S16AS24A^ although it is not as severe as Subito^ΔNT^.

**Figure 4:**
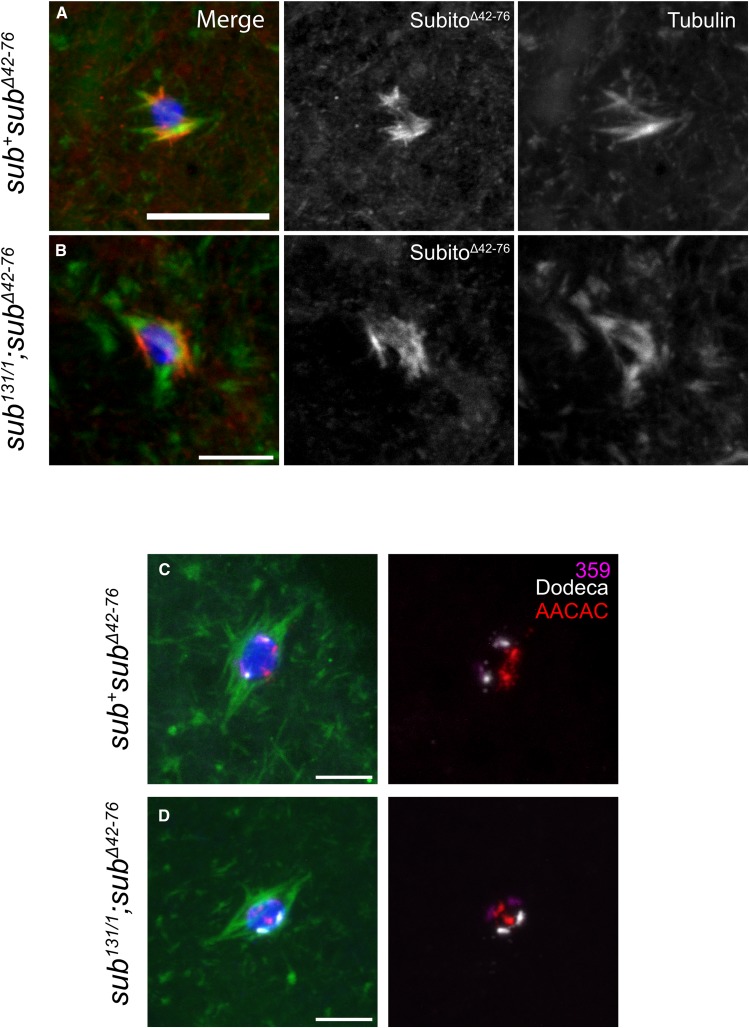
The poorly conserved domain 42-76 is required for establishing bipolarity and homolog bi-orientation. A,B. Localization of Subito^Δ(42-76)^ in *sub^+^* or *sub^131/1^* null background oocytes. Oocytes are stained for DNA (blue), HA (red), tubulin (green). Single channels for HA and tubulin are shown in white. Scale bars are 5 µm. C,D. FISH in the indicated genotypes using probes for the X (359 in magenta), 2^nd^ (AACAC in red) and 3^rd^ chromosomes (Dodeca satellite in white). (n= 28, 17, 18 and 12 respectively).

**Figure 5:**
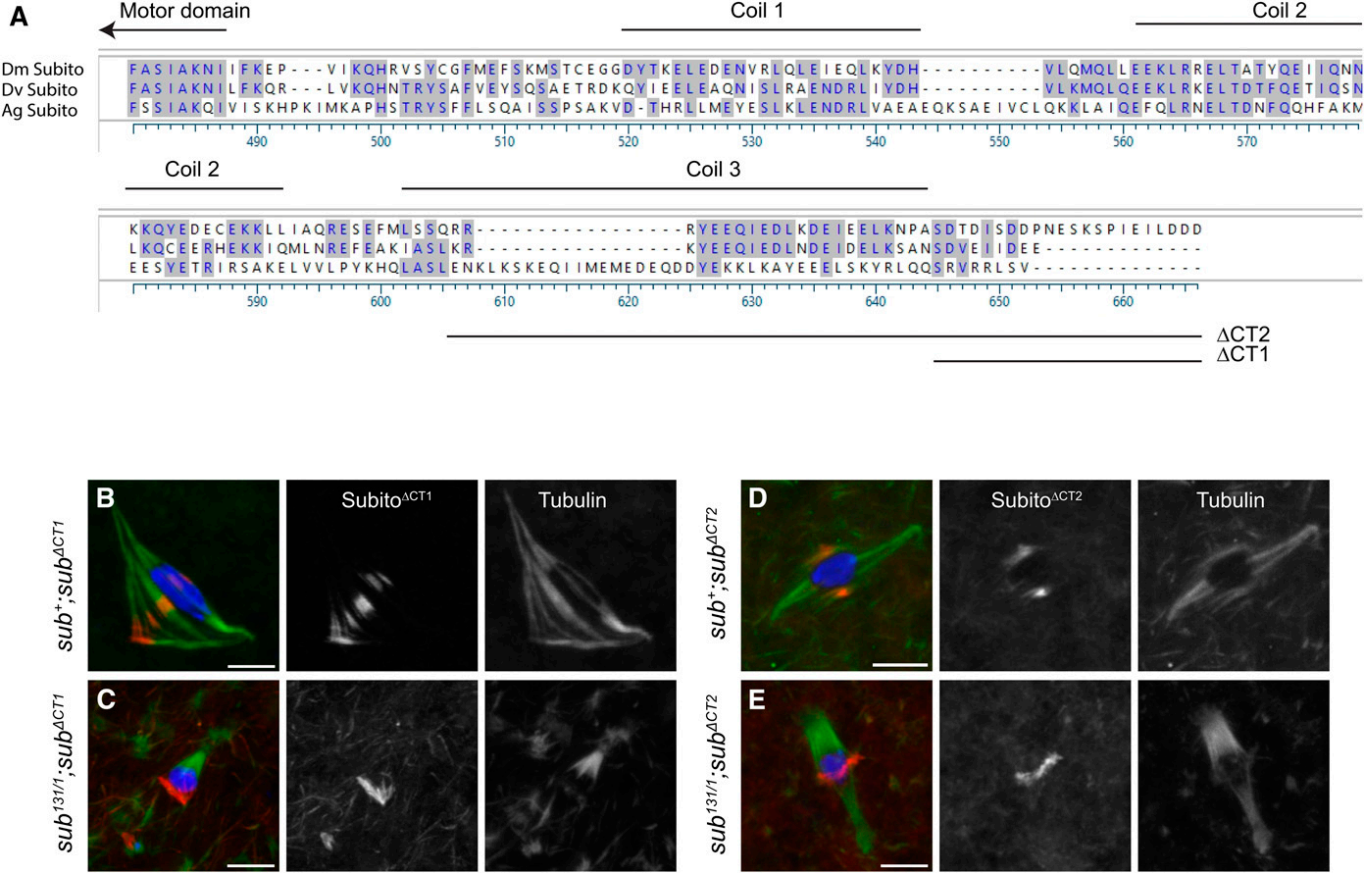
The C-terminal domain of Subito is required to establish bipolarity of the central spindle. A. Alignment of the C-terminal of Subito in *Drosophila melanogaster*, *Anopheles gambiae* and *Drosophila virilis* showing the deleted regions and the conserved coiled coil. B-E) Localization of indicated transgenes in *sub^+^* or *sub^131/1^* null background. Oocytes are stained with DNA (blue), HA (red), tubulin (green). Single channels for HA and tubulin are snoshown in white. Scale bars are 5 µm. Panel C shows an example of a monopolar spindle. (n= 30, 12, 27, 5, 25 respectively).

**Figure 6:**
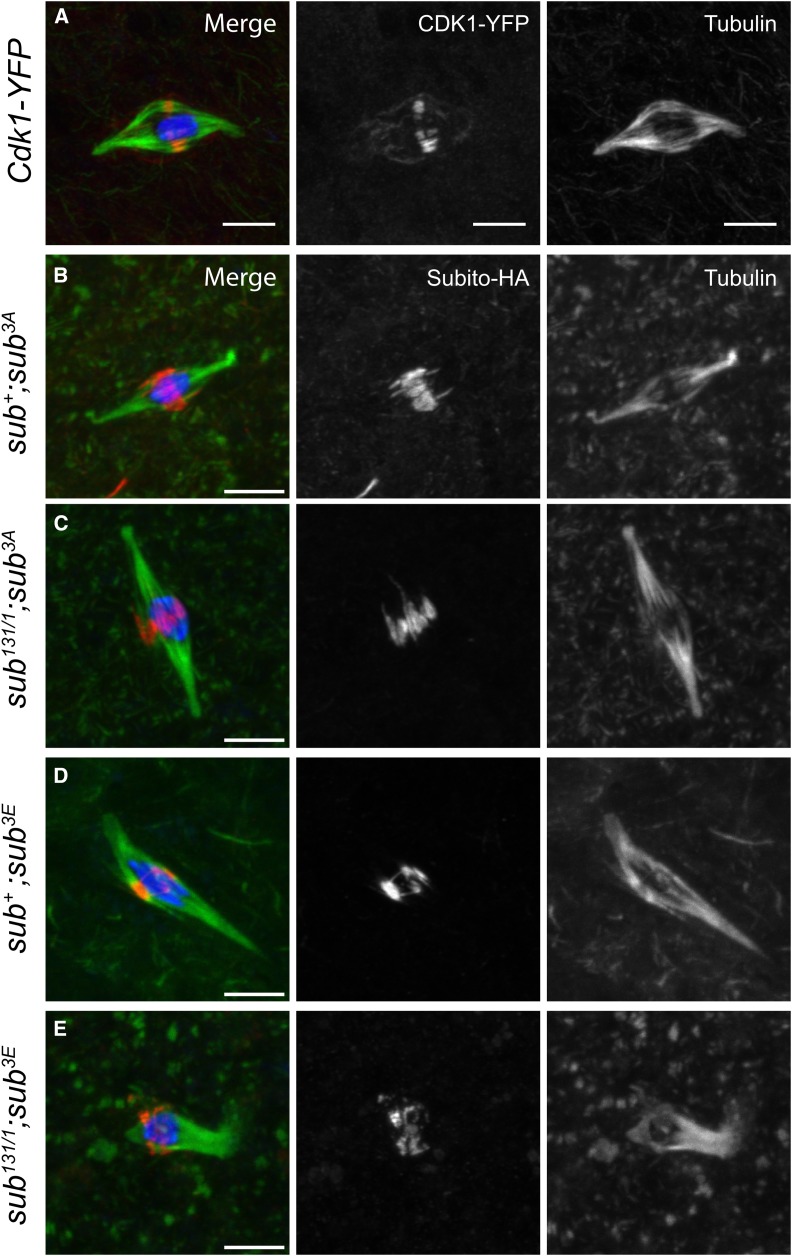
Cdk1 phosphorylation may inhibit Subito activity at meiotic metaphase I. A. Oocyte expressing CDK1-YFP (Ayeni *et al.* 2014). B-D. Localization of Subito-3A or Subito-3E mutants in *sub^+^* or *sub^131/1^* null background (n = 7, 10 and 15 respectively). Oocytes are stained with DNA (blue), HA or GFP (red), tubulin (green). Single channels for HA or GFP and tubulin are shown in white. Scale bars are 5 µm.

**Figure 7:**
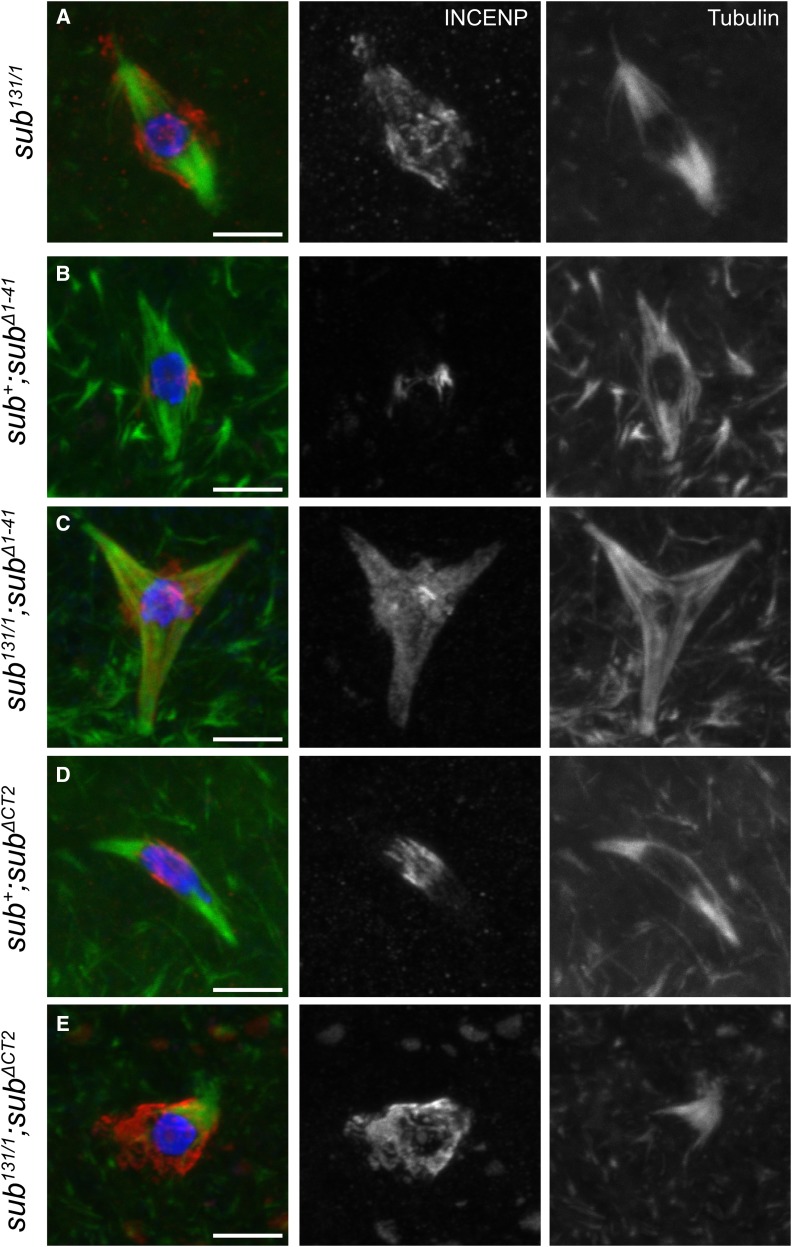
The N and C terminal regions of Subito are independently required for INCENP localization to the meiotic central spindle. All panels show oocytes stained with DNA (blue), INCENP (red in merge, white in single channel) and tubulin (green in merge and white in single channel). A-E. INCENP localization in oocytes expressing Subito^Δ(1-41)^ or Subito^ΔCT2^ in *sub^+^* or *sub^131^*^/^*^1^* background respectively. INCENP is diffusely localized in C and E, compared to the compact ring localization to the central spindle in B and E, which also have wild type Subito present in oocytes. (n= 11, 9, 5 and 10 respectively).

All Drosophila stocks and DNA clones are available upon request. Representative images are presented within the article and complete imaging data sets are available upon request. Fertility and nondisjunction data for *subito* transgenes in a wild-type background are in Table S1 (in File S1). The percentages of oocytes in each category of spindle morphology determined cytologically is in Table S2 (in File S1). Examples of these spindle categories are in Figure S2. Western blots showing oocyte expression of each mutant transgene is shown in Figure S1, Figure S3, and Figure S5. Cytology of mutants not shown in Figures 1-7 are shown in Figure S4 and Figure S5. Supplementary material has been uploaded to figshare https://doi.org/10.25387/g3.5952370.

## Results

### Mutational analysis of the Subito N-terminal domain

We have previously shown that deletion of the N-terminal domain of Subito (the first 76 amino acids before the motor domain), tagged with GFP, results in ectopic spindles, suggesting the N-terminus restricts the activity of the kinesin to the chromosomes (Jang *et al.* 2007). For mutational analysis of its regulatory sequences, full length *subito* cDNA and mutant variants were cloned into a vector fused to three copies of the HA epitope tag. This construct with the N-terminal deletion (ΔNT) resulted in ectopic bundles of microtubules in the ooplasm away from the chromosomes, although it was not as severe as the GFP tagged Subito version of this mutant (Jang *et al.* 2007) (Figure 1A,B). By western blot, the *GFP - sub* fusions often had a higher level of Subito protein compared to *HA - sub* fusions (Figure S1). The ectopic spindle phenotype was not observed with expression of full length Subito, suggesting that the N-terminal tag or overexpression was not the cause of the ectopic spindle phenotype, but the severity depends on expression levels.

Within the N-terminal domain, the first half (amino acids 1-41) is conserved in other insects while there is less conservation in the second half of this domain (Figure 1C,D). To determine if these regions contribute to the regulation of Subito in meiosis, we created a series of deletions and substitutions. For all the experiments, *UASp:sub* transgenes were expressed in the female germline using *P{GAL4*::*VP16-nos.UTR}MVD1*, which has *GAL4* fused to the *nanos* promoter and induces the expression of *UASp* regulated transgenes in the female germline (Rørth 1998). The activity of these transgenes was genetically measured using two maternal phenotypes. First, Subito is required for spindle bipolarity and bi-orientation in meiosis. To assay for this meiotic function, we tested if expression of the mutant transgenes could rescue the X-chromosome non-disjunction phenotype of the hypomorphic mutation *sub^1794^* (41%, Table 1). In addition, the ability of each mutant protein to localize and organize a bipolar meiotic spindle was determined. In these cytological experiments, we also asked if any of the mutants recapitulated the *sub^ΔNT^* phenotype of ectopic bundles of microtubules in the ooplasm. Second, Subito is required for pronuclear fusion and embryogenesis (Giunta *et al.* 2002). To assay for this embryonic function, we tested if expression of the mutant transgenes could rescue the sterile phenotype of the null mutant genotype *sub^131^ /sub^1^* (Table 2). In addition, we tested if the mutants had effects on fertility or nondisjunction in the presence of wild-type Subito (Table S1). Previous work has shown that mutations within the motor domain often have dominant phenotypes (Jang *et al.* 2007).

**Table 1:** Rescue of a *sub^131/1794^* mutant with mutant transgenes

**Transgene**	**Regular Progeny**	**Non-disjunction Progeny**	**Female Parent**	**Progeny/Female Parent**	**NDJ (%)**
*sub^131^/ sub^1794^*	470	121	270	2.19	40.9
*sub^HA^*	3494	4	76	46.1	0.2
*sub^Δ(1-4)1^*	1944	138	323	6.45	13.3
*sub^Δ(24-33)^*	3040	14	255	11.98	0.9
*sub^S16AS24A^*	1818	11	150	12.19	1.2
*sub^Δ(42-76)^*	1471	76	212	7.30	9.8
*sub^ΔCT1^*	1745	182	153	12.6	17.3
*sub^ΔCT2^*	501	153	187	3.5	37.9
*sub^3E^*	3257	176	76	47.5	9.8

The first row shows non-disjunction in *sub^131^/sub^1794^* background. All other rows indicate the transgene expressed using a *P{Gal4*::*VP16-nos.UTR}MVD1* in the *sub^131^/sub^1794^* background. The females were crossed to *yw/B^s^Y* males to assess non-disjunction. *sub^HA^* is the full length wild-type transgene. ·For each mutant, multiple transgenes were tested but only one representative result is shown. *sub^Δ(1-21)^* was not tested because it does not localize in wild type background and has no dominant phenotypes.

**Table 2:** Rescue of a *sub^131/1^* null mutant with N-terminus mutant transgenes

**Transgene**	**Female Parent**	**Total progeny**	**Progeny/Female Parent**	**Nondisjunction (%)**
*sub^HA^*	36	1376	38.3	0.4
*sub^Δ(1-41)^*	288	175	0.6	33.1
*sub^Δ(1-21)^*	28	0	0	ND
*sub^Δ(24-33)^*	96	252	2.6	0.8
*sub^S16A^*	30	264	8.8	1.5
*sub^S24A^*	40	400	10.0	0.2
*sub^S16AS24A^*	216	46	0.2	4.3
*sub^Δ(42-76)^*	148	717	4.8	16.2
*sub^ΔCT1^*	56	50	0.9	30.5
*sub^ΔCT2^*	77	0	0	ND
*sub^3A^*	88	1298	14.8	0.8
*sub^3E^*	52	1011	20.6	12.1

ND = not determined due to sterility.

Each transgene was expressed using a *P{Gal4*::*VP16-nos.UTR}MVD1* in a *sub^131/1^* null mutant background, which is sterile. Resultant female progeny were tested for fertility by crossing to *y w/B^s^Y* males to assess fertility. *sub^HA^* is the full length wild-type transgene. ·For each mutant, multiple transgenes were tested but only one representative result is shown.

### Antagonistic elements within the first 41 amino acids in Subito regulate central spindle assembly in oocytes

The N-terminal domain was split with two deletions (*sub^Δ(1-41)^ and sub^Δ(42-76)^*) (Figure 1C,D). A smaller deletion that only included the less conserved first 21 amino acids in *sub^Δ(1-41)^* was also constructed (*sub^Δ(1-21)^*). Expression of Subito^Δ(1-41)^ in a wild-type background did not show dominant phenotypes (Table S1) or produce the ectopic spindle phenotype like the deletion of the entire N-terminus. However, Subito^Δ(1-41)^ was only able to partially rescue non-disjunction of *sub^1794^/sub^131^* (Table 1), and sterility of *sub^131/1^* mutant females (Table 2), indicating that this domain is important for meiotic as well as embryonic functions of Subito.

In contrast, Subito^Δ(1-41)^ localized to the central spindle in oocytes, similar to wild type (Figure 2C, D), in the presence or absence of wild-type protein. In the absence of wild-type protein, Subito^Δ(1-41)^ did not rescue the spindle defects of a *sub^131/1^* mutant oocyte (Figure 2D and Figure S2B, Table S2). There are numerous spindle defects in a *sub^131/1^* mutant oocyte, including polarity defects, lack of a central spindle and other microtubule organization defects which we have separated into different categories (Figure 2A, Figure S2). Subito^Δ(1-41)^ localizes to the central spindle and hence can organize some interpolar microtubules, but other defects like fraying, multipolarity, knobbed or split spindles persist (Figure S2B, Table S2). Taken together with the result that Subito^Δ(1-41)^ partially rescues non-disjunction and cannot rescue sterility, we conclude that assembly of the meiotic central spindle is necessary but not sufficient for Subito function and that domain 1-41 is essential for the function of the central spindle.

Surprisingly, Subito^Δ(1-21)^ failed to localize even though the protein was expressed in *sub+*; *sub^Δ(1-21)^* oocytes (Figure 2E,F, Figure S3), explaining why it fails to rescue the sterility and central spindle defects in *sub^131/1^*; *sub^Δ(1-21)^* oocytes (Table 2, Figure 2F). To explain the failure of Subito^Δ(1-21)^ to localize to the spindle while the larger deletion Subito^Δ(1-41)^ does, we suggest that there are antagonistic regulatory elements in the N-terminus: a positive regulatory element in the first 21 amino acids and a negative regulatory element in the next 20 amino acids. In the absence of the first 21 amino acids, sequences within the second 20 amino acids may prevent localization; hence deletion of both elements restores the spindle localization activity to the protein.

### Conserved serines within the N-terminal domain may help restrict Subito activity in oocytes to the vicinity of the chromosomes

To investigate the antagonistic regulatory properties within the 1-41 region, additional mutations of amino acids conserved in other *Drosophila* species were generated (Figure 1). The mutation *sub^Δ24-33^* deletes the most conserved amino acids in this domain, including S24 which is phosphorylated in *Drosophila* Kc167 cells (Bodenmiller *et al.* 2007). The Subito^Δ24-33^ protein rescued the non-disjunction phenotype of *sub^1794^/sub^131^* (Table 1) but not the sterility phenotype of *sub^131/1^* (Table 2), suggesting that amino acids 24-33 are required for mitosis and/or pronuclear fusion but not meiosis. Consistent with this result, Subito^Δ(24-33)^ localized normally to the central spindle in a wild-type or *sub^131/1^* background. Expression in a wild-type background did not result in dominant fertility or nondisjunction phenotypes (Table S1) and did not show the ectopic spindle phenotype (Figure 3A, B). Thus, the conserved amino acids 24-33 are not required for central spindle assembly and meiotic function but are indispensable for embryogenesis. This supports the idea that there are activities of Subito that are important for embryonic development but not meiosis.

In addition to S24, there is a second conserved serine (S16) within region 1-41 (Figure 1D). To determine the role of these serine residues in Subito, we substituted S16 and S24 to alanines and generated single (*sub^S16A^* and *sub^S24A^*) and double (*sub^S16AS24A^*) mutants. All three mutant proteins (Subito^S16A^, Subito^S24A^ and Subito^S16AS24A^) localized to the central spindle, indicating that these residues are not required for localization (Figure 3, Figure S4). Subito^S16AS24A^ was able to rescue the non-disjunction phenotype of *sub^1794^/sub^131^* although it had reduced fertility in this cross compared to *sub^Δ24-33^* (Table 1). Interestingly, while Subito^S16AS24A^ did not rescue the sterility phenotype of *sub^131/1^*, Subito^S16A^ and Subito^S24A^ were able to confer a partial rescue of fertility (Table 2), suggesting the two serines have a redundant function.

Surprisingly, expression of *sub^S16AS24A^* in wild type oocytes also resulted in a high frequency of frayed spindles (45%) (Figure 3C). Localization of Subito^S16AS24A^ in *sub^131/1^*; *sub^S16AS24A^* oocytes was abnormal and not restricted to the ring shape characteristic of the wild type protein (Figure 3D). Furthermore, bundles of microtubules were observed in the ooplasm similar to but reduced as compared to *sub^ΔNT^* expressing oocytes (Figure 3E, F). These results suggest that S16 and S24 promote the restriction of Subito localization to the central spindle. This function may be less important for meiosis but is critical for pronuclear fusion or mitosis. In the absence of S24 or S16, we did not observe ectopic bundles in the ooplasm, suggesting S16 and S24 are partially redundant for this function, and that these two serine residues may be important for regulating the activity of Subito to ensure it is active with microtubules only in the vicinity of the chromosomes.

### The non-conserved half of Subito N-terminus has dominant effects on meiotic spindle organization and is required for homolog bi-orientation

The *sub^Δ(42-76)^* mutant deletes the second less conserved half of the N-terminal domain. Surprisingly, spindle organization defects were observed in *sub^+^;sub^Δ(42-76)^* oocytes (64% bipolar spindles, 21% frayed, 11% tripolar and 4% monopolar) (Figure 4A). This dominant phenotype shows that the mutant protein has an activity that can interfere with the function of the wild type protein, leading to spindle fraying and polarity defects. These dominant phenotypes also complicate the interpretation of rescue experiments. Thus, it is not surprising that expression of Subito^Δ(42-76)^ did not full rescue the spindle defects in *sub^131/1^*; *sub^Δ(42-76)^* oocytes (52% bipolar, 36% multipolar/frayed and 12% tripolar, Figure 4B) when compared to the *sub^131/1^* null oocytes. Subito^Δ(42-76)^ localization was relatively normal within the central spindle, demonstrating that this protein localizes to the spindle, is able to organize some interpolar microtubules, but still has some spindle defects. Similarly, expression of Subito^Δ(42-76)^ did not fully rescue non-disjunction in *sub^1794^/sub^131^* (Table 1) and failed to rescue sterility of a *sub^131/1^* null mutant (Table 2). These results show that, although poorly conserved, amino acids 42-76 are required for both mitotic and meiotic functions of the kinesin (Table 1). In the absence of these amino acids, the protein has a gain of function that causes spindle defects in the presence of the wild-type protein.

Even though *sub^+^;sub^Δ(42-76)^* oocytes had abnormal spindles, we did not detect elevated levels of nondisjunction in a wild-type background (Table S1). To determine if the abnormal spindles in *sub^+^;sub^Δ(42-76)^* oocytes were associated with bi-orientation defects, we performed FISH using probes to the heterochromatin region AACAC on the second chromosome, Dodeca on the third and 359 on the X. Wild type oocytes usually bi-orient their chromosomes efficiently with minimal mis-orientation, as published earlier (Radford *et al.* 2012; Das *et al.* 2016). However, in *sub^+^;sub^Δ(42-76)^* oocytes the X chromosomes were mis-oriented in 30% (Figure 4C) and AACAC and Dodeca in 33% of oocytes and this usually correlated with spindle polarity defects. As expected, the bi-orientation defect in *sub^131/1^*; *sub^Δ(42-76)^* oocytes were similar to a *subito* null mutant (75% for the X and the 2^nd^ chromosome and 66% for the 3^rd^) (Figure 4D). To reconcile the observations of a dominant bi-orientation defect with genetic results that did not show a defect in X-chromosome segregation in *sub^+^;sub^Δ(42-76)^* oocytes (Table S1), it is possible that a dynamic meiotic spindle corrects the defects prior to anaphase I, or only the euploid meiotic products are selected to fuse with the sperm and give offspring.

### C-terminal domain is required for the activity of Subito but not its localization

The Subito C-terminal domain is capable of binding to the spindle independently of the motor domain (Jang *et al.* 2007). Like other kinesins, the C-terminal domain contains predicted coiled-coil domains that may be important for interactions with microtubules or other proteins (Mason and Arndt 2004). Additionally, mass spectrometry analysis of *Drosophila* embryos has shown that the C-terminus of Subito contains several phosphorylation sites (Zhai *et al.* 2008; Hilger *et al.* 2009). A previous study of a mutant with a complete deletion of the C-terminus was not informative because the protein was unstable in ovaries and not detected on western blots (Jang *et al.* 2007). However, a motor-only construct, lacking the N- and C-terminal domains but retaining the motor and linker region, was detected on western blots (Figure S5). This protein failed to localize to the spindle, suggesting that Subito requires both the motor and C-terminal domains to localize to the spindle.

To investigate the function of the C-terminal domain and the predicted phosphorylation sites, we constructed two deletions. The first mutation, *sub^ΔCT1^*, deleted the last 22 amino acids, including most of the C-terminal domain amino acids shown to be phosphorylated by mass spectrometry, while leaving the coiled-coil domains intact (Figure 5A). The second larger mutation, *sub^ΔCT2^*, deleted the last 43 amino acids, which in addition to the region deleted in *sub^ΔCT1^*, also deletes the last of the three predicted coiled-coil regions (Figure 5A).

Genetic analysis revealed normal levels of X chromosome non-disjunction when either mutant was expressed in a wild-type background (Table S1). Expression of *sub^ΔCT1^* or *sub^ΔCT2^* did not rescue the nondisjunction of the *sub^1794^/sub^131^* mutant (Table 1) or the sterility of the *sub^131/1^* null mutant (Table 2). The larger deletion had a more severe defect in nondisjunction, even more severe than any N-terminal domain mutant. The *sub^ΔCT2^* mutant was also the only one to exhibit chromosome loss; 83% of the aneuploid progeny were from nullo-X oocytes. This suggests that, not only did homologous chromosome fail to bi-orient, they also were not segregated into any meiotic products.

Even though these mutants lacked wild-type function, both Subito^ΔCT1^ and Subito^ΔCT2^ localized to the central spindle in a wild-type or mutant background (Figure 5C, E). Consistent with the genetic results, however, neither mutant rescued the spindle defects of *sub^131/1^* null mutant oocyte (Figure 2A, Figure S2B). Both *sub^131/1^*; *sub^ΔCT1^* and *sub^131/1^*; *sub^ΔCT2^* oocytes had high percentages of spindle defects (Figure S2, Table S2) with a surprisingly high level of monopolar spindles (57% and 32% respectively), compared to *sub^131/1^* null mutant oocytes (3% monopolar spindles, Figure 5C,E). The high frequency of monopolar spindles cold help explain the high frequency of chromosome loss in the *sub^ΔCT2^* mutant. These results show that the C-terminal domain, including the coiled coil region, are necessary to keep the spindle poles separated and maintain bipolarity but is not required for central spindle localization.

### Putative CDK1 phosphorylation sites may inhibit Subito activity at metaphase I

Subito colocalizes with Cyclin B and CDK1 on the meiotic spindle (Swan and Schupbach 2007) (Figure 6A). This observation presents a paradox because high levels of CDK1 activity during mitotic metaphase inhibit MKLP2 interactions with the microtubules (Hümmer and Mayer 2009; Kitagawa *et al.* 2014). Dephosphorylation of CDK1-dependent sites promotes MKLP2-mediated translocation of the CPC to the anaphase midzone. Studying the role of CDK1 is problematic because it is required for entry into meiosis (Bourouh *et al.* 2016). Therefore, to investigate the role of CDK1 phosphorylation of Subito, we identified three possible proline-directed CDK1 phosphorylation sites in Subito (S207, T457, S620) and mutated all of them to either alanine (3A) or glutamate (3E).

These mutants were tested for the rescue of the *sub^1/131^* sterility phenotype. The *sub^3A^* allele rescued most aspects of the *sub* null phenotype. The Subito^3A^ protein localized normally to the central spindle, bipolar spindles were formed, and nondisjunction was drastically reduced (Figure 6B,C, Table 1, Table 2). There was a small reduction in fertility. In contrast, while the *sub^3E^* mutant protein localized in the *sub^1/131^* background (Figure 6C), there were spindle defects in the oocytes (Figure 6D,E, Figure S2, Table S2). Consistent with these results, there was reduced fertility (Table 2) and elevated nondisjunction (Table 1) in the rescue experiments. These results suggest that dephosphorylation of the predicted CDK1 sites is important for the meiotic functions of Subito.

### The Subito N- and C-terminal domains are required to restrict INCENP localization to the meiotic central spindle

The defective Subito proteins in the majority of mutants we characterized localized to the central spindle. These mutant proteins may be capable of localizing to spindle microtubules, but their interaction with components of the central spindle is hindered. Indeed, in the absence of Subito, INCENP localization is abnormal and not restricted to the central spindle (Figure 7A) (Radford *et al.* 2012). Therefore, we examined INCENP localization in two mutants, *sub^Δ(1-41)^* and *sub^ΔCT2^*,both of which localize normally but do not rescue meiotic or mitotic functions of *sub* null mutants and have substantial spindle polarity defects. When mutant transgenes were expressed in a *sub^+^* background, INCENP was restricted to a ring at the central spindle (Figure 7B, D). In contrast, INCENP localization was diffuse and spread out along the all spindle microtubules in *sub^Δ(1-41)^* and *sub^ΔCT2^* oocytes (Figure 7C, E). These results show that, although these mutant Subito proteins localize, they are unable to organize the central spindle, leading to a diffuse INCENP localization. An important function of kinesin 6 proteins like Subito is to regulate the localization of the CPC, which is essential for meiotic and mitotic spindle assembly and function.

## Discussion

Most eukaryotes have several classes of kinesin proteins that are defined by the conserved sequence of their motor domains. It is unclear, however, what parts of each kinesin contribute to their unique functions; how much is due to motor domain and how much is due to the less conserved flanking domains (Hammond *et al.* 2010; Welburn 2013)? Several non-exclusive mechanisms have been found to regulate kinesin motor localization and activity. Autoinhibition may involve a folded state where the C-terminal tail inhibits the motor. Similarly, some kinesins are inactive in the monomeric state but becomes active through clustering and dimerization (Okada *et al.* 1995; Rashid *et al.* 2005; Hammond *et al.* 2009). A kinesin could be regulated by interactions with other spindle associated proteins or phosphorylation. For MKLP2, a higher-order clustered structure is important for microtubule binding and function (Kitagawa *et al.* 2014).

What specifies central spindle localization of kinesin 6 proteins is not known. We showed previously that the motor domain is required for localization of Subito to the central spindle (Jang *et al.* 2007) while the C-terminal domain can independently bind to spindle microtubules. In this study we have investigated the roles of the divergent N- and C-terminal domains of the kinesin 6 Subito. Our results demonstrate that the entire N-terminal domain and part of the C-terminal domain are dispensable for central spindle localization, but the motor domain itself is not sufficient (Table 3). It is likely that central spindle localization depends on cooperation between the motor domain and at least part of the C-terminal domain. Mutations in the N- or C- terminal domains fail to rescue motor functions in spite of their normal localization. These observations suggest that both domains are required for motor regulation and there are multiple inputs that regulate the activity, in addition to the localization, of Subito.

**Table 3:** Summary of phenotypes and functions for the different mutant Subito transgenes

**Subito mutant**	**Dominant effects**	**Rescue of mitotic function (sterility)**	**Rescue of meiotic function (NDJ)**	**Localizes to central spindle**	**Required for MT organization in oocytes**	**Required for CPC localization**
***subito^Δ(1-41)^***	No	No	Partial	Yes	Yes	Yes
***subito^Δ(1-21)^***	No	No	No	No	Yes	-^1^
***subito^Δ(24-33)^***	No	No	Yes	Yes	No	—
***subito^S16AS24A^***	Yes (frayed spindles, ectopic)	No	Yes	Yes but delocalized	Yes, and for restricting Subito to CS ^1^	—
***subito^Δ(42-76)^***	Yes (spindle pole defects)	No	Partial	Yes	Yes	—
***subito^ΔCT1^***	No	No	No	Yes	Yes	Yes
***subito^ΔCT2^***	No	No	No	Yes	Yes	—
***subito^3A^***	No	Yes	Yes	Yes	No	—
***subito^3E^***	No	No	No	Yes	Yes	—
***Subito^1794^***	No	Yes ^3^	No ^3^	Yes ^3^	No ^3^	—

1–- = not tested. 2 - CS = Central spindle, 3 - data from (Giunta *et al.* 2002).

### Antagonistic regulatory elements within the N terminal domain

Deletion of the first 21 amino acids abolished localization to the meiotic central spindle. Curiously, a larger deletion of the first 41 amino acids restored central spindle localization (Table 3). While the 1-21 deletion may disrupt folding of the protein while 1-41 does not, based on the analysis of the *Drosophila* kinesin 14 NCD (Beaven *et al.* 2017), we suggest that the region between amino acids 22-41 contains a negative regulator of localization. NCD is inhibited by phosphorylation at S96 by an unknown kinase, but this is blocked by phosphorylation at S94 by Aurora B. We suggest an inhibitory phosphorylation occurs between amino acids 21-41, the effects of which can be blocked by phosphorylation between amino acids 1-21. The most likely inhibitory site is S24, which is known to be phosphorylated (Bodenmiller *et al.* 2007), while activating phosphorylation could occur at S16 (likely due to close proximity to S24), S3 or S7. Beaven *et al.* suggest this is a mechanism to limit NCD activity to the vicinity of the chromosomes where Aurora B activity is high. Our previous observation that a deletion of the N-terminus (Jang *et al.* 2007), or mutation of both S26 and S24, causes ectopic microtubule bundling in the ooplasm is consistent with a similar mechanism regulating Subito.

While amino acids 1-41 may function to restrict Subito activity to the chromosomes, the less conserved amino acids 42-76 has a different function. Loss of these amino acids has dominant effects on spindle morphology that effects meiotic functions of establishing bipolarity and bi-orientation. These results are consistent with a “rigor” phenotype that was initially expected, but not observed, from mutation of the ATP binding domain (Jang *et al.* 2007). Thus, this region of the N-terminal domain may modulate or negatively regulate the activity of the protein. The large number of serine residues (11 between 42 and 90), a conserved feature of this region (Figure 1), may provide a mechanism to fine-tune activity with phosphorylation events.

### The C-terminal domain regulates activity and localization to microtubules

The C-terminal domain contains three coiled-coil motifs. In both Subito and the vertebrate homolog MKLP2, the C-terminus is thought to be important for microtubule binding (Jang *et al.* 2007; Kitagawa *et al.* 2014). The kinesin 7 CENP-E also has an elongated coiled coil or stalk that is required for efficient microtubule-kinetochore activity (Vitre *et al.* 2014). One of the deletions studied here deletes the last coiled-coil, suggesting the first two coiled-coil domains plus the motor domain are sufficient for central spindle localization. This may include interactions with the CPC and dimerization with another Subito molecule (see Figure S1). Central spindle localization appears to require two microtubule interactions, one through the motor domain and the second through the C-terminal domain.

The region after the coiled-coils is required for full Subito activity but not its localization. In MKLP2, the region after the coiled-coils interacts with lipids, a function important for abscission at the end of cytokinesis (Fung *et al.* 2017). The role of this region in oocyte meiosis, where interactions between the metaphase I spindle and the nuclear envelope are not obvious, is unclear. Like we proposed for the N-terminal domain, this region contains known phosphorylation sites which may regulate Subito activity (Zhai *et al.* 2008; Hilger *et al.* 2009). Alternatively, this region could be involved in protein-protein interactions with other spindle-associated proteins. An interesting possibility is that this region is required to silence interactions between the N- and C-terminal domain that repress activity. In this case, we would predict that sequences in the C-terminal domain interact with the negative regulatory element in 22-41 region of the N-terminal domain.

Results in mammalian cells have shown that MKLP2 is negatively regulated by CDK1 phosphorylation (Hümmer and Mayer 2009; Kitagawa *et al.* 2014). In *Drosophila*, kinesin 6 Pavarotti is also negatively regulated by CDK1 phosphorylation (Goshima and Vale 2005). Mutations to eliminate phosphorylation of Subito by CDK1 had minimal effects on meiosis or fertility. Instead, we observed that a phosphomimetic mutant had defective chromosome segregation. This suggests that limiting CDK1 phosphorylation of Subito is important for meiosis. The reason why CDK1 and Subito both occupy the meiotic metaphase I central spindle and the role of phosphorylation is mysterious.

### Relationship Between Subito mediated meiotic central spindle assembly and chromosome bi-orientation

Our results have shown that elements in the N- and C-terminus are required for regulating the activity but not localization of Subito. We have also found evidence of separation of function mutations. These are not simply partial loss of function mutations, but represent defects in specific activities that are more important to meiosis or embryonic development. Some mutants most severely affect meiosis but not embryonic function, such as *sub^1794^* (Giunta *et al.* 2002)) and the *3E* mutant. Conversely, there are mutants with more severe defects in embryogenesis than meiosis (such as *sub^(24-33)^* and *sub^S16AS24A^*) (Table 3).

One of the regulatory activities of Subito affected by these mutants is restricting CPC localization to the central spindle in meiotic metaphase I. Thus, although the truncated motors can bind microtubules, they cannot perform a function like directing CPC localization. This is the first evidence for a specific role of the N- and C-terminal domains in regulating CPC localization. Hence, the failure to rescue phenotypes like chromosome bi-orientation and nondisjunction could be attributed to a defect in CPC regulation. Indeed, an *Incenp* mutant that fails to restrict CPC activity to the central spindle has dominant meiotic nondisjunction phenotype (Radford *et al.* 2012).

Another possibility is that the defective Subito proteins in these mutants fail to execute a function required of the central spindle. For example, a structural study of MKLP2 suggested that Kinesin 6 proteins could function to organize or sense tension of microtubules, rather than have a transport function (Atherton *et al.* 2017). Non-transport roles have also been suggested in other contexts, including for the NOD kinesin 10 motor that is required for *Drosophila* meiosis (Cui *et al.* 2005; Cochran *et al.* 2009; Richard *et al.* 2016). We envision mutants that are proficient in assembling antiparallel microtubule bundles could be defective in how they are organized or the ability to respond to tension. This activity could occur in the context of “bridging fibers”, which were originally described as microtubule fibers in mammalian cells that bundled with kinetochore fibers and connect sister kinetochores. They connect mitotic metaphase sister kinetochores that are separated because they are under tension (Kajtez *et al.* 2016; Vukusic *et al.* 2017). We have also observed similar structures that contain Subito in *Drosophila* mitotic cells (Cesario *et al.* 2006). In meiosis, the central spindle contains bundles of MTs between bi-oriented homologous centromeres. Adopting the bridging fiber model to meiosis, we suggest that bi-orientation depends on homologous centromeres interacting with the same bundle of central spindle MTs. Indeed, in prometaphase oocytes, it is often possible to observe pairs of homologous centromeres connected by a bundle of antiparallel MTs, which we propose are bridging fibers. Properly modulated Subito activity may be required for homologous chromosomes to separate from each other along the interpolar microtubules of bridging fibers.
